# Assessing the effects of common topical exposures on skin bacteria associated with atopic dermatitis

**DOI:** 10.1002/ski2.41

**Published:** 2021-05-07

**Authors:** C. R. Castillo, M. E. Alishahedani, P. Gough, P. P. Chaudhary, M. Yadav, J. Matriz, I. A. Myles

**Affiliations:** Epithelial Therapeutics Unit, National Institute of Allergy and Infectious Disease, National Institutes of Health, Bethesda, Maryland, USA

## Abstract

**Background:**

While patients and families struggling with atopic dermatitis (AD) have documented concerns for a contributory role of skin care products in AD pathology, nearly all the skin microbiome studies to date have asked participants to avoid topical products (such as soaps or select medications) for the preceding days to weeks prior to sample collection. Thus, given the established role of the microbiome in AD, the interactions between topical exposures, dysbiosis and AD remains underrepresented in the academic literature.

**Objectives:**

To address this knowledge gap, we expanded our previous evaluations to test the toxicological effects of a broader range of common chemicals, AD treatment lotions, creams and ointments using both health- and AD-associated strains of *Roseomonas mucosa* and *Staphylococcus* spp.

**Methods:**

Use of in vitro culture techniques and mouse models were deployed to identify chemicals with dysbiotic or pre-biotic potential. A proof-of-concept study was subsequently performed in healthy volunteers to assess global microbiome shifts after exposure to select chemicals using dermatologic patch testing.

**Results:**

Numerous chemicals possessed antibiotic properties, including many not marketed as anti-microbials. Through targeted combination of potentially beneficial chemicals, we identified combinations which promoted the growth of health-associated isolates over disease-associated strains in bacterial culture and enhanced microbe-specific outcomes in an established mouse model of AD; the most promising of which was the combination of citral and colophonium (often sold as lemon myrtle oil and pine tar). Additional studies would likely further optimize the combination of ingredients use. Similar results were seen in the proof-of-concept human studies.

**Conclusions:**

Our results could offer a systematic, multiplex approach to identify which products carry dysbiotic potential and thus may guide formulation of new topicals to benefit patients with AD.

## 1 | INTRODUCTION

Atopic dermatitis (AD) is an inflammatory disease of the skin associated with reduced quality of life and increased risk for developing asthma, allergic rhinitis and food allergies.^1^ The microbiome is increasingly recognized as both a significant contributor to AD pathology^2^ and a potential therapeutic target.^3–6^ However, because previous research protocols have asked participants to avoid skin care products for the days to weeks prior to sample collection,^7–10^ the current literature connecting dysbiosis to AD cannot comment on the potential contribution of topical product exposures to AD pathology. This knowledge gap at the intersection of topical exposures, dysbiosis and AD is a documented source of anxiety for the patients with AD and parents of children with AD struggling to optimize their skin care regimens.^11,12^ The knowledge gap also affords market space for products that claim to ‘balance the microbiome’ either without any supporting data or based solely on in vitro inhibition of *Staphylococcus aureus*.^13^

Herein we expand upon prior work establishing that chemicals commonly found in personal care products could theoretically cause dysbiosis through disruption of healthy microbial balance.^5,14^ We screened a larger number of compounds and developed an in vitro predictive index for the potential to preference the growth of disease- or health-associated strains of Gram-positive *Staphylococcus* spp. In addition, we investigated the impact of topical products on the growth of *Roseomonas mucosa*, a commensal strain of Gram-negative bacteria that we have shown offers therapeutic benefit in children and adults through its unique production of lipids which induce tissue regeneration and inhibit *S. aureus* growth.^4–6^

Although our work dose not present definitive combinations of topical ingredients, through targeted mixture of potentially beneficial chemicals, we did identify combinations which promoted the growth of health-associated isolates over disease-associated strains in bacterial culture and enhanced microbe-specific outcomes in mice. In a proof-of-concept study in healthy volunteers, we found similar results by performing genomic evaluation after dermatologic patch testing against these topicals. The results of this investigation present a systematic approach to identify the skin products that carry dysbiotic potential and may guide formulation and optimization of new topicals for benefiting patients with AD.

## 2 | METHODS

### 2.1 | Bacterial collection and identification

Bacterial isolates were collected as previously described under the IRB approved protocol after written and oral consent was provided.^20^ Briefly, two FloqSwabs (Copan) moistened in sterile phosphate-buffered saline (PBS; Corning Cellgro) were rubbed on the subject’s skin at the antecubital fossa and volar forearm vigorously for 15–30 s. For patients with atopic dermatitis, sampling was done at these sites from affected lesional skin if present. One swab was placed into a 15-ml conical tube (Corning Life) with 2 ml of sterile Hank’s balanced salt solution (HBSS; Sigma-Aldrich) containing vancomycin (300 ug/ml) and amphotericin B (5 μg/ml; Sigma-Aldrich) to inhibit the growth of Gram-positive bacteria and fungi. The remaining swab was placed into a 15-ml conical tube containing 2 ml of R2A (Reasoner’s 2A) broth (Teknova) with similar concentrations of vancomycin and amphotericin B. The tubes, with swabs left in place, were then incubated at 32°C with constant shaking for 48–72 h before plating 100 μl from each tube onto an R2A agar plate (Remel). Colonies were then taken for species identification by mass spectrometry using matrix-assisted laser desorption/ionization-time of flight (MALDI-TOF) analysis. Bacterial protein extraction for MALDI-TOF MS using the BioTyper (v3.1, Bruker Daltonics Inc.) was performed by the NIH Clinical Center microbiology lab using previously described methods,^36^ instrument settings and calibration.^37,38^ BioTyper identification was supplemented by additional mass spectra profiles provided by several NIH developed databases.^36,39,40^ All *R. mucosa* isolates used for subsequent studies were verified by MALDI-TOF analysis. Isolates were selected for topical exposure testing based on their impact in in vitro models of AD,^6^ mouse models,^6^ and based on the therapeutic benefits seen in clinical trials.^4,5^

### 2.2 | Chemical selection and testing

Chemicals were chosen to form a broad representation of exposures that AD patients might encounter. Several common bathing therapies for AD were tested, including seawater, Dead Sea salt solution and citric acid baths.^16^ A variety of parabens, a common preservative, were tested as well as the Chemotechnique NAC-80 patch test kit. The patch test kit features a range of common preservatives, additives and other chemicals meant to simulate typical environmental exposure levels.

The broth-soluble chemicals were dissolved in R2A or BHI broth media and diluted to the concentrations described in the literature. Chemicals tested include 480 mM NaCl, 54.14 mM MgSO4, parabens (methyl, ethyl propyl, butyl and benzyl), ZnO, Zn, soap, iodine, 5% Dead Sea salt solution, 0.005% bleach and 75% ethanol as a control. In a 96-well plate, 100 μl of chemical was combined with 100 μl of 1/50 dilute bacteria. One row of wells was reserved for 200 μl of broth media and one row of wells was reserved for 100 μl of 1/50 dilute bacteria and 100 μl of broth media. The 96-well plates containing strains of *R. mucosa* were incubated at 32°C for 24 h and 120-rpm shaking. The 96-well plates containing strains of *Staph* spp. were incubated at 37°C for 3 h and 120-rpm shaking. Following the incubation period, absorbances were collected at 600 nm using a BioRad Benchmark Plus plate reader. Absorbance of the bacteria incubated with the chemical was compared to absorbance of the bacteria incubated in broth media and percent change of bacterial growth was calculated.

Chemicals insoluble in broth (Chemotechnique Diagnostic NAC-80 patch test kit and lotions) were incubated with bacteria on agar plates. 100 μl of 1/50 dilute *R. mucosa* was plated on R2A agar plates and 100 μl of 1/50 dilute *Staph.* spp was plated on Remel blood agar plates. Approximately 1 ml of chemical was applied to a round glass coverslip and placed in the centre of the agar plate. Plates of *R. mucosa* were incubated at 32°C for 48 h and plates of *Staph.* spp were incubated at 37°C for 24 h. Following incubation, zone of inhibition was measured with Neiko digital calipers.

### 2.3 | Isolate selection and culturing

Three healthy volunteer (HV) and three disease-associated (AD) strains of *R. mucosa* were cultured in 5 ml Reasoner’s 2A (R2A) broth media for 24 h at 32°C. Three HV and three AD strains of *Staph* spp. were cultured in 5 ml blood heart infusion (BHI) broth media for 24 h at 37°C. The cultured bacteria were vortexed and diluted to 1/50 in 5 ml broth media. Selection was as previously described.^4,6^

### 2.4 | Growth index calculation

To quantify the chemicals’ effect on microbial growth balance, a growth index was created. Rather than measuring *R. mucosa* or *Staph* spp. growth individually, it synthesizes them into a measurement that assesses how the chemical might affect conditions for growth on the skin. The growth index aggregates *R. mucosa* and *Staph* spp. growth measurements (either zone of inhibition or percent growth) and calculates the overall balance between Gram− and Gram+ growth. A negative growth index value denotes conditions where *Staph* spp. is able to outcompete *R. mucosa* and vice versa for a positive growth index value.

### 2.5 | Mix derivation

Colophonium was ground into powder using a Qiagen TissueLyser LT and steel bead. Colophonium powder, fusidic acid and butyl paraben were dissolved in molecular biology-grade distilled H_2_O and mixed with Cetaphil, Gold Bond lotions or Vaseline in a Fisher Scientific Bead Mill 4 for 90 s at speed 5. Lemon myrtle oil (Tea Tree Therapy) was mixed into indicated products by stir.

### 2.6 | Patch testing

Six total healthy volunteers were enrolled in an IRB-approved study NCT03921515 and exposed to the topical chemicals via patch testing. Patch testing was used because the commercially available systems represent real-world concentrations for each product as used in skin care products. After mixing with Vaseline as above, 12 total wells of a Finn Chamber patch test (SmartPractice) were used. Three wells were Vaseline diluent alone, two were blank and the remaining wells contained the indicated chemical. Each location was randomized so that the relative positions of each chemical to each other were different for each participant. Comparisons between diluent control and chemical-infused patches were made via 16S and ITS analyses. Each participant was instructed to avoid all topical skin care products for the 24 h before and during the patch testing experiment.

### 2.7 | Microbial DNA extraction from human skin

Microbial DNA was extracted from the host as previously described,^4^ and extracted using the Qiagen DNA Microbiome kit (Hilden) using the Qiagen QIAcube as per manufacturer instructions. Isolated genomic DNA was quantified with Qubit 2.0 DNA HS Assay (ThermoFisher).

### 2.8 | Targeted microbiome analysis

Library prep and sequencing were performed by CosomosID. For 16S V1–V3, libraries were prepared using the Illumina 16S Metagenomic Sequencing kit (Illumina, Inc.) according to the manufacturer’`s protocol. The V1–V3 region of the bacterial 16S rRNA gene sequences were amplified using the primer pair designed to amplify that specific region with Illumina adapter overhang nucleotide sequences at 5’ end. The full-length primer sequences are:27F: 5’ TCGTCGGCAGCGTCAGATGTGTATAAGAGACAG-[AGAGTTTGATCCTGGCTCAG]534R: Reverse overhang: 5’ GTCTCGTGGGCTCGGAGATGTGTATAAGAGACAG-[ATTACCGCGGCTGCTGG].

Amplicon polymerase chain reaction (PCR) was performed to amplify target gene out of input DNA templated from each respective sample. Briefly, each 25 μl of PCR reaction contains 12.5 ng of sample DNA as input, 12.5 μl 2x KAPA HiFi HotStart ReadyMix (Kapa Biosystems) and 5 μl of 1 μM of each primer. PCR reactions were carried out using the following reaction conditions: an initial denaturation step performed at 95°C for 3 min followed by 25 cycles of denaturation (95°C, 30 s), annealing (55° C, 30 s) and extension (72°C, 30 s), and a final elongation of 5 min at 72°C in a thermal cycler. PCR product was cleaned up with Mag-Bind RxnPure Plus magnetic beads (Omega Bio-tek). A second index PCR amplification, used to incorporate barcodes and sequencing adapters into the final PCR product, was performed in 25 μl reactions, using the same master-mix conditions as described above. Cycling conditions were as follows: 95°C for 3 min, followed by 8 cycles of 95°C for 30 s 55°C for 30 s and 72°C for 30 s. A final, 5-min elongation step was performed at 72°C. The libraries were normalized with Mag-Bind Equi-Pure Library Normalization Kit (Omega Bio-tek) and then pooled. The pooled library was qualified and quantified using an Agilent 2200 TapeStation and sequenced (2 × 300 bp paired-end read setting) on the MiSeq (Illumina). Because testing supplies were not sterile, sequencing of supplies was performed and those signatures were subtracted from the participant samples prior to analysis. The remaining reads in the samples were calculated for relative abundance and generation of 3D PCA plots were done by the CosmosID application.

For fungal diversity studies in our 50 ng of isolated genomic DNA was used to amplify via PCR with proprietary primers (ITS1 and ITS2). All primers were synthesized by Integrated DNA Technologies covering ITS1 and ITS2 regions. Specific primer selection and design (Admera Health, LLC) were chosen to achieve comprehensive taxonomic coverage, elimination of spike-in to gain maximal data. Final library quantity was assessed by Qubit 2.0 (ThermoFisher), and quality was assessed by TapeStation D1000 ScreenTape (Agilent Technologies Inc.).

Sequences of both the ITS primers used are as follows: ITS1-F ACCTGCGGARGGATCA; ITS1-R GAGATCCRTTGYTRAAAGTT; ITS2-F GTGAATCATC-GARTCTTTG; ITS2-R TCCTCCGCTTATTGATATGC.

Illumina® 8-nt dual indices were used. Equimolar pooling of libraries was performed based on QC values and sequenced on an Illumina MiSeq V2 (Illumina) with a read length configuration of 2 × 250 bp.

### 2.9 | Mouse model

Female or male mice aged 6–8 weeks (age and sex matched within each experiment) were treated in the MC903 model as previously described.^5^ MC903 was applied to both ears on days 10 to 0, when applied 10^4^ colony-forming units of USA300LAC strain of *S. aureus* or of HV1 strain of *R. mucosa* were applied to both ears on days 0 and 1, then a PBS solution containing lemon myrtle oil (1% v/v in water) with colophonium (5% w/v) was applied to both ears on days 1 and 2. Ears were imaged and harvested on day 7. For experiment using a combination of bacterial isolates, MC903 model dermatitis was induced as before. For 2 consecutive days (days 0 and 1), mice were given a topical application of a 1:1:1 mixture of 5e5 CFU of *Rm*HV, CONS-HV and *Sa*-AD immediately followed by application of Vaseline, a mixture of the patch test reagents for fragrance mix II and colophonium, or the patch test paraben mix. Ear were imaged and harvested on day 5.

### 2.10 | Scratch assay

Scratch assay was performed as previously described using the HaCaT keratinocyte cell line purchased from American Tissue Culture Collection (ATCC). Experiments were performed as previously described. Briefly, 100,000 cells were seeded in 24-well plates and allowed to adhere to the culture plate overnight. Cells challenged with bacteria were stimulated overnight prior to scratch; cells challenged with chemicals were stimulated 4 h prior to scratch using the Autoscratch (BioTek). Cells were placed in the Cytation 5 (BioTek) at 37°C with 5% CO_2_; images and quantitation were performed by the Scratch App (BioTek).

## 3 | RESULTS

### 3.1 | Common topical products display antibiotic properties

**FIGURE 1:**
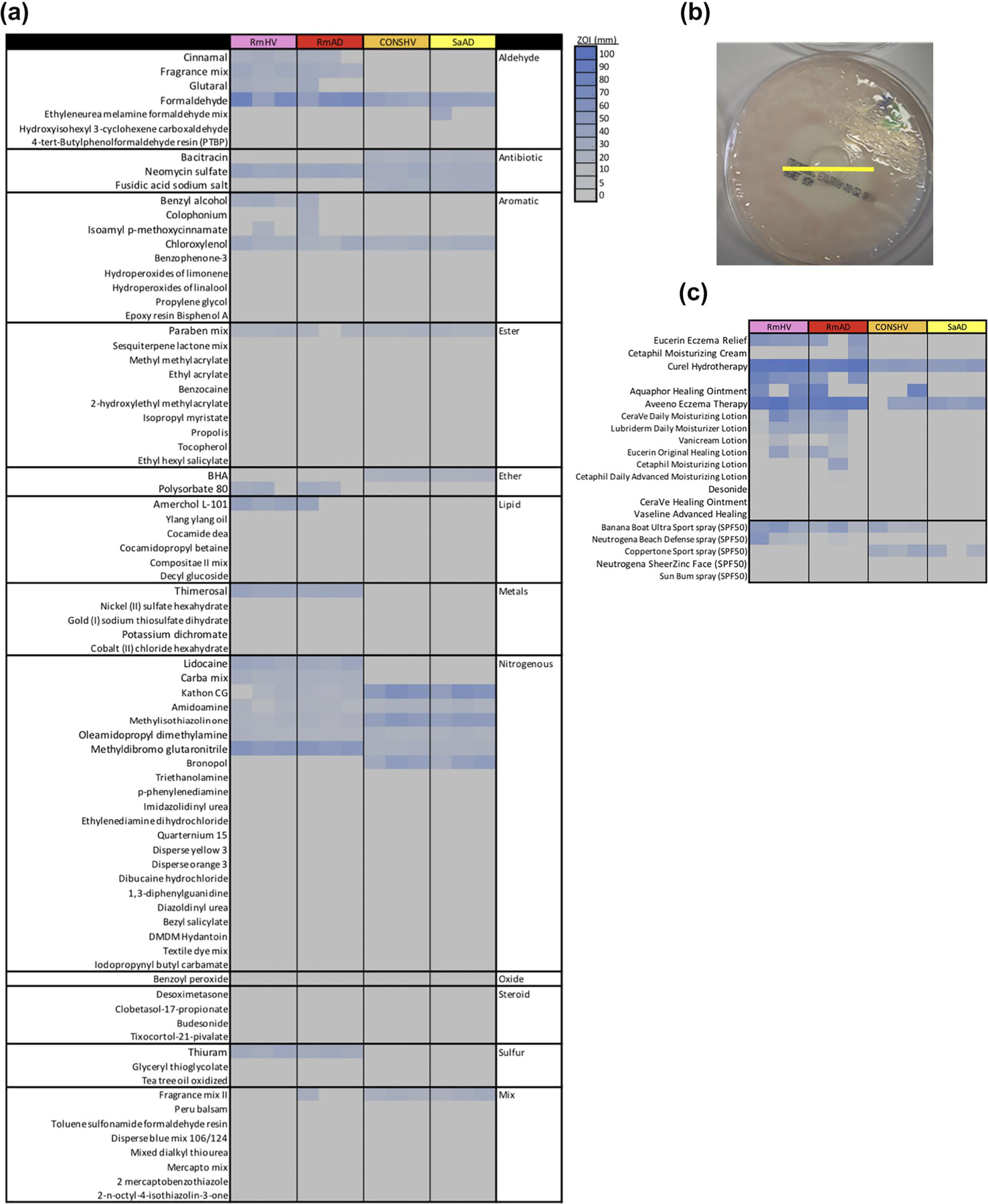
Common chemical exposures show antibiotic like properties. (a) Zone of inhibition (ZOI) for chemicals contained in NAC80 patch test kit. (b) Sample image showing how ZOI were measured; yellow line added to indicate measured diameter. (c) ZOI for select lotions marketed as eczema treatments. Data shown for three isolates each of *R. mucosa* from health volunteers (*Rm*HV) or patients with atopic dermatitis (*Rm*AD), coagulase-negative *Staphylococcus* from HV (CONS-HV) or *S. aureus* from AD patients (SaAD). Results are representative of four independent experiments

Chemicals from a clinically approved patch test kit (North American Comprehensive 80 from Chemotechnique Diagnostics) were tested against select bacterial isolates in an agar-based protocol mirroring those for assessing antibiotic resistance. Three representative isolates each were tested for: *R. mucosa* collected from the skin of healthy controls (*Rm*HV) or patients with atopic dermatitis (*Rm*AD); coagulase-negative *Staphylococcus* spp. from healthy volunteers (CONS-HV) and isolates of *S. aureus* collected from patients with atopic dermatitis (*Sa*AD). Twenty-seven of the 80 compounds tested demonstrated antibiotic properties as indicated by a visible zone of inhibition (ZOI; Figure 1a,b). Chemicals that were roughly classified as aldehydes, ethers and metals were more likely to carry antibacterial effects than other classifications. However, variations were seen within chemical classes, chemical structures as well as between isolates of the same species (Figure 1a; Figure S1). Over the counter topicals containing some of the ingredients of interest demonstrated similarly variable results despite many of the tested products being marketed to patients with ‘eczema’ (Figure 1c).

### 3.2 | Products preference growth of disease- over health-associated strains of commensal bacteria

**FIGURE 2:**
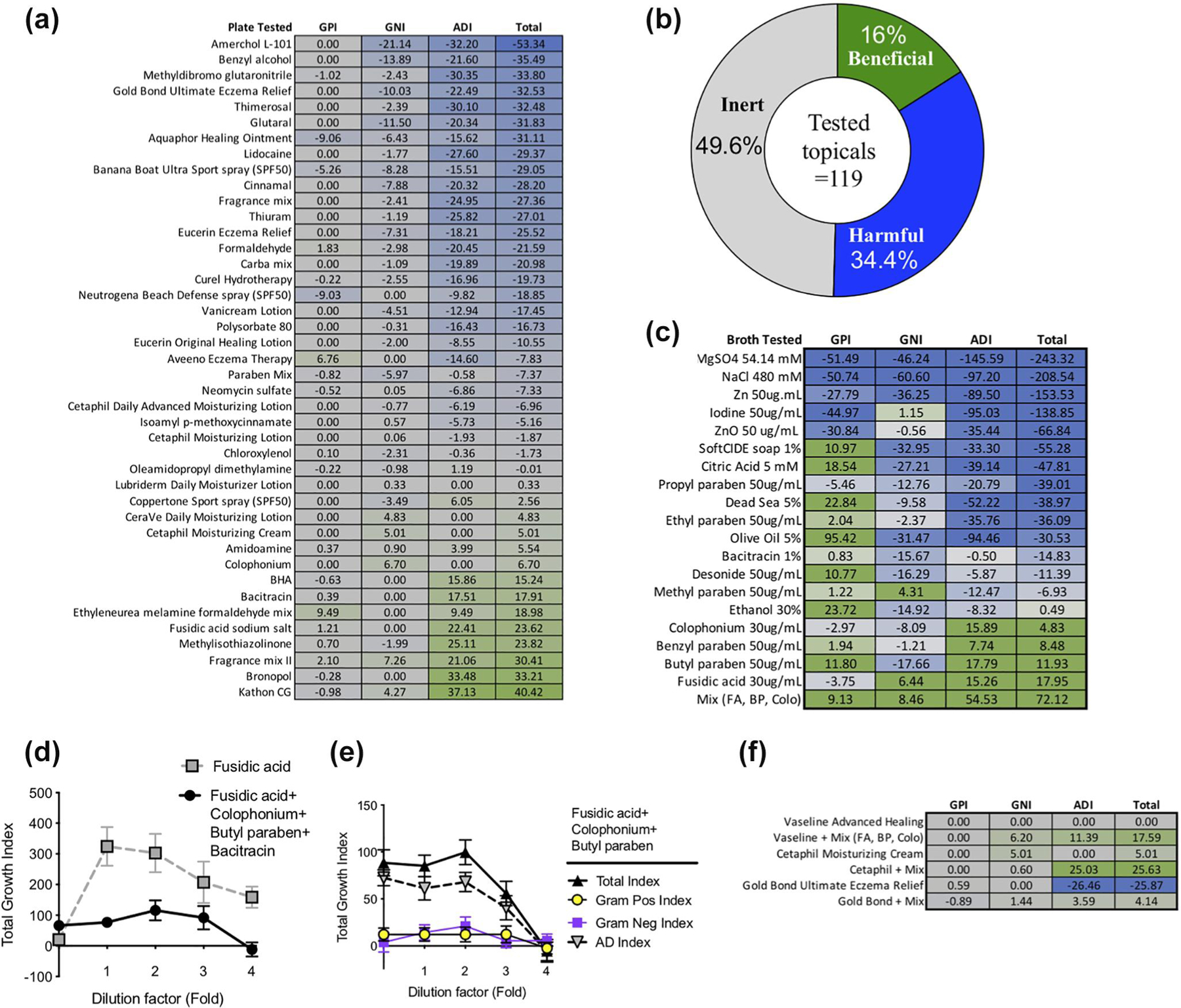
Targeted selection of topical products can influence in vitro microbiome growth. (a) Predictive indexes as determined by the relative inhibition for health-versus disease-associated isolates of *Staphylococcus* spp. and *Roseomonas mucosa* on agar culture plates. Calculations for Gram-positive (GPI), Gram-negative (GNI) and atopic dermatitis index (ADI). Green indicates a predicted beneficial impact; blue indicates those with predicated negative impact on microbiome balance as it would relate to the risk of developing atopic dermatitis. (b) Overview of the predicted indexes for all 119 chemicals/topicals tested in agar plate format. Inert defined as total index of zero, beneficial defined as total index >0 and harmful defined as total index <0. (c) Predictive indexes as determined by the relative inhibition for health-versus disease-associated isolates of *Staphylococcus* spp. and *R. mucosa* in broth culture. (d) Total predictive index for fusidic acid alone (starting at 300 mg/ml) and a combination of fusidic acid (starting concentration 25 μg/ml), colophonium (35 μg/ml), butyl paraben (125 ng/ml) and bacitracin (14 pg/ml) with indicated number of 10-fold dilutions. (e) Total, Gram-positive, Gram-negative and AD index for a combination of indicated chemicals with same concentrations as in panel A without bacitracin. (f) Predictive index calculated on agar cultures for indicated topicals alone or combined with Plate agar indexes derivation for Vaseline, Cetaphil or Gold Bond topicals with and without addition of fusidic acid (FA; 453 μg/ml), colophonium (Colo; 833 μg/ml), and butyl paraben (BP; 3.3 pg/ml). Data representative of (a, c–f), or a combination of (b) five independent experiments

Multiple indexes were developed to quantify the impact of each chemical on the in vitro balance of bacterial growth: the Gram-positive index (GPI) comprised the average of three representative of CONS-HV ZOI minus the average ZOI for three AD *S. aureus* strains; the Gram-negative index (GNI) represented the average *Rm*HV ZOI less the average *Rm*AD ZOI; the AD Index (ADI) represented the average *Sa*AD ZOI less the average *Rm*HV ZOI; and the total index was the sum of the GPI, GNI and ADI. Under this derivation, chemicals that selectively inhibited *Rm*HV and CONS-HV would carry a negative score and be expected to skew the skin microbiome towards higher burden of disease-associated isolates. Among the non-inert topicals, a majority had a net negative index (Figure 2a,b). Much of the total index was comprised of the ADI, which indicates that many chemicals would provide greater inhibition against health-associated strains of *R. mucosa* than disease-associated strains of *S. aureus*.

Notable chemicals in the plate agar assay were tested in broth culture to attempt to determine potential growth enhancement. In addition to those that could be solubilized, we selected various topicals with literature reports of therapeutic benefit for AD and/or eczema.^15–17^ In the broth assay, many chemicals preferentially inhibited disease-associated *S. aureus* compared to health-associated CONS-HV but still demonstrated an overall negative total index (Figure 2c). Broth cultures also allowed for assessment of interactions between ingredients. For example, while colophonium, bacitracin, fusidic acid and butyl paraben each had predictive beneficial effects independently (Figure 2a), these beneficial effects were not present when the chemicals were combined (Figure 2d). A combination of colophonium (42 μg/ml), fusidic acid (23 μg/ml) and butyl paraben (1.7 pg/ml) synergized for the predicted positive effect on in vitro microbial balance in broth culture (Figure 2e; compared to independent values found in Figure 2c). This combination of chemicals also improved the overall predicted indexes of Vaseline (an inert ointment), Cetaphil (the cream with the best growth index) and Gold Bond (the cream with the worst growth index; Figure 2f).

### 3.3 | Combination of colophonium, butyl paraben and fusidic acid improved mouse model outcomes

**FIGURE 3:**
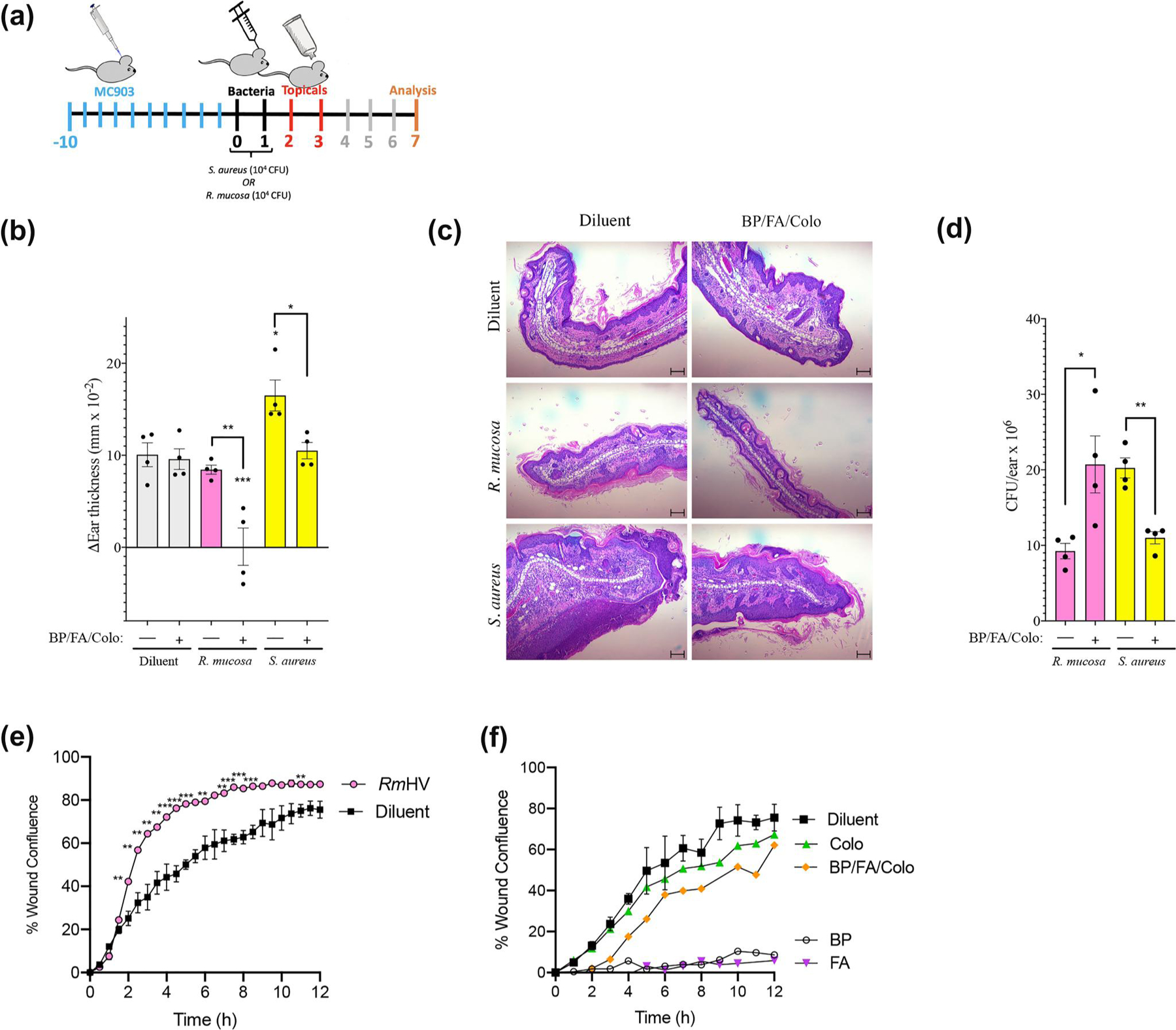
Targeted selection of topical products improves outcomes in mouse models of atopic dermatitis. (a) Diagram of protocol for mouse studies presented in figure. Dermatitis was induced using daily MC903 on the ears for Day 10 through 0. Mice were treated once daily with diluent or 10^4^ colonies of *R. mucosa* or *S. aureus* for days 0–1. Either diluent control or topical butyl paraben (BP; 3.3 pg/ml), fusidic acid (FA; 453 μg/ml), combined with colophonium (Colo; 833 μg/ml) in PBS was applied to the ears once daily on days 2–3. (b) Change in ear thickness between Day 0 and Day 7. (c) Representative histology for median mouse per group from Day 7; scale bars, 100 μm. (d) Colony forming units (CFU) per ear for mice treated with of *R. mucosa* or *S. aureus* with and without topical treatment. (e) Wound closure over time for HaCaT cell line keratinocytes stimulated with diluent or *R. mucosa* from healthy volunteers (*Rm*HV) at multiplicity of infection (MOI) of 10. (f) Wound closure over time for HaCaT cells stimulated with 5 μM concentrations of indicated chemicals. Data represents three independent experiments. Dots indicate individual mice. *p < 0.05; **p < 0.01; ***p < 0.001 as determined by ANOVA with Bonferroni’s adjustment and displayed as mean ± SEM. PBS, phosphate buffered saline

In a mouse model of AD (Figure 3a), topical application of the combination of colophonium, butyl paraben and fusidic acid enhanced the therapeutic benefits (Figure 3b,c) and growth (Figure 3d) of an otherwise sub-therapeutic dose of *Rm*HV. Similarly, this combination countered the harmful impacts and growth of *S. aureus* (Figure 3b–d). The combination alone did not impact modelled outcomes (Figure 3b,c) and thus appeared to enhance the microbe-specific phenotypes for treatment rather than directly modulate host responses.

### 3.4 | Topical products had variable impacts on models of wound healing

Our previous mechanistic studies demonstrated that the benefits of *R. mucosa* treatment are in part mediated by induction of wound healing pathways, notably epithelial-to-mesenchymal transition (EMT).^6^ One in vitro model of EMT is the ‘scratch assay’ wherein cells are grown to confluence, ‘scratched’ with a sterile pipette tip, and monitored as the cells on the leading-edge attempt to ‘heal’ the wound by filling in the space via migration and/or proliferation.^6,18^ As previously described,^6^ *R. mucosa* enhances the modelled healing in the scratch assay (Figure 3e). Colophonium did not significantly impact scratch closure time in human keratinocytes (Figure 3f). However, butyl paraben and fusidic acid in isolation directly inhibited scratch closure, but not when in combination with colophonium (Figure 3f).

### 3.5 | Combination of lemon myrtle oil and colophonium improved predicted impact of topical products

**FIGURE 4:**
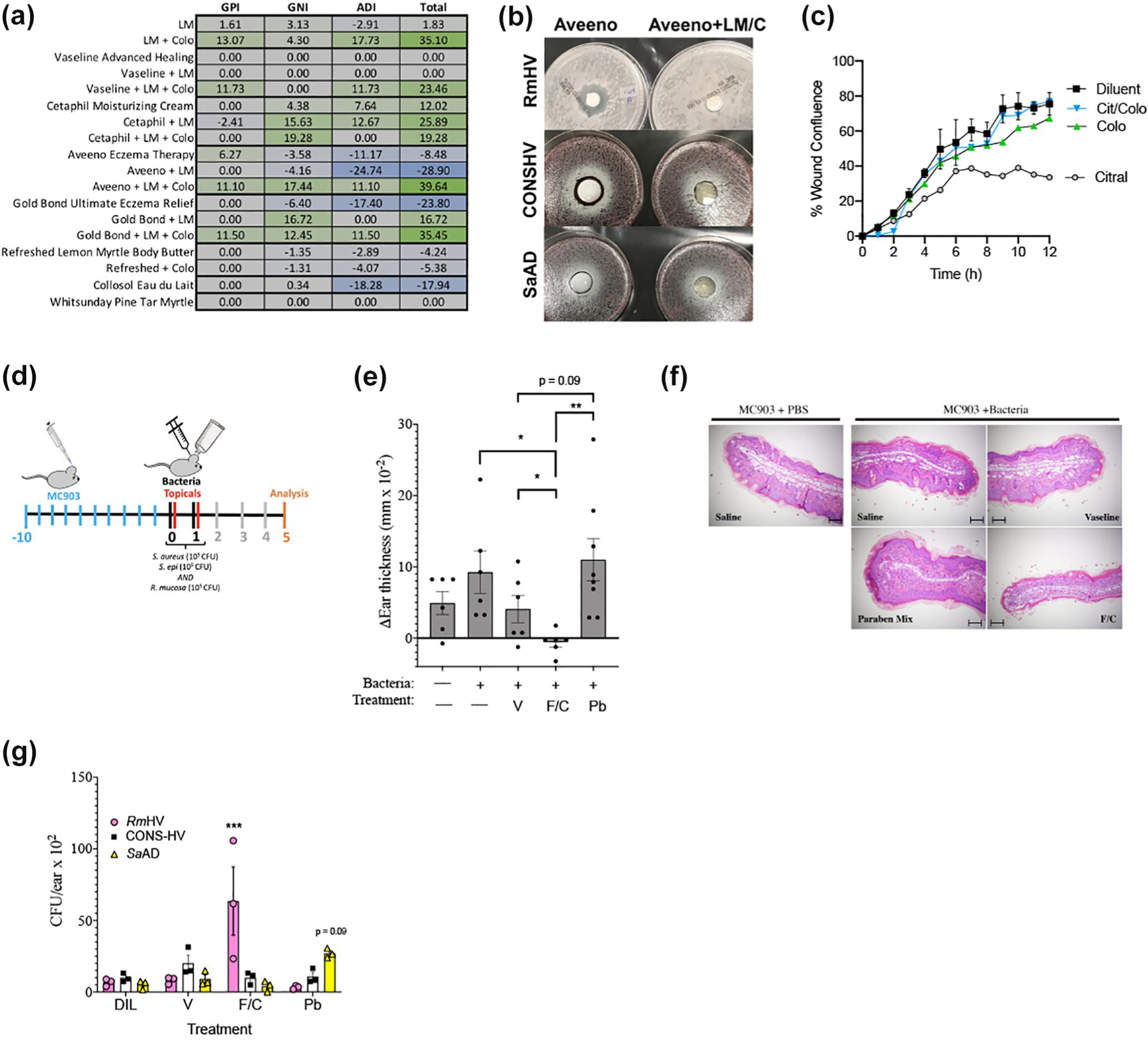
Targeted selection of natural products improves outcomes in culture and mouse models of atopic dermatitis. (a) Predictive indexes for indicated topicals alone or combined with lemon myrtle oil (LM; 1% v/v) and colophonium (Colo; 833 μg/ml). (b) Representative photo of plate from select isolates for Aveeno Eczema Therapy (left) or Aveeno with lemon myrtle oil and colophonium (right). (c) Wound closure over time for HaCaT cells stimulated with 5 μM concentrations of citral alone or citral in equal 5 μM concentration with colophonium (Colo = colophonium; Cit/Colo = combination). (d) Diagram of protocol for mouse studies presented in figure. Dermatitis was induced by MC903 as before. On days 0–1, topical application of a 1:1:1 mixture of 10^5^ colony forming units (CFU) of *R. mucosa* from healthy volunteers (*Rm*HV), coagulase-negative *Staphylococcus* from HV (CONS-HV) and *S. aureus* from AD patients (*Sa*AD) daily for 2 days (bacteria). Immediately after each bacterial application mice were treated with Vaseline (V), a mixture of the patch test reagents for fragrance mix II and colophonium (F/C; final concentrations 7% and 10% w/v, respectively), or the patch test paraben mix (Pb, 16% w/v). Chemicals were applied to the ears the same day as the bacterial mix to prevent the differing growth curves of *Staphylococcal* spp. and *R. mucosa* from impacting microbial balance before topical exposure. (e) Change in ear thickness between Day 0 and Day 5, (f) representative histology from the median mouse per group from Day 5 scale bars, 100 μm, and (g) CFU per ear for each indicated bacterial isolate are shown. Data represents three independent experiments. Dots indicate individual mice. *p < 0.05; **p < 0.01; ***p < 0.001 as determined by ANOVA with Bonferroni’s adjustment and displayed as mean ± SEM

Fragrance mix II also demonstrated favourable indexes in the agar plate assay (Figure 2a). This mix contains six different chemicals: α-hexylcinnamicaldehyde, citral, citronellol, coumarin, farnesol and lyral. Citral, is a natural oil found in lemon and orange peels, lemongrass (*Cymbopogon*) and lemon myrtle (*Backhousia citriodora*). We opted to focus on citral given it is known to inhibit *S. aureus* without inducing spontaneous resistance.^19^ Lemon myrtle oil (1% v/v in water) alone had only moderately positive predictive indexes and variable impacts when combined with select topical products (Figure 4a). However, the combination of 1% lemon myrtle oil and colophonium (5% w/v) demonstrated a highly positive predicted index in aqueous solution and improved the indexes of selected topicals (Figure 4a,b). We tested one topical product which professed to contain lemon myrtle oil (Refreshed Lemon Myrtle Body Butter; Tea Tree Therapy Inc); however, it did not demonstrate a positive predictive index and could not be rescued by the addition of colophonium (Figure 4a). We identified two products which claim to contain both citral and colophonium: a makeup remover with both citral-containing parfum and colophonium (eau du lait; Collosol) which presented a negative total index; and a balm that included both lemon myrtle oil and colophonium-containing pine tar (Pine Tar Myrtle Balm; Whit-sunday) which was inert against all strains tested (Figure 4a). In the scratch assay, the combination of citral and colophonium had no impact on wound healing time, despite the finding that citral alone was inhibitory (Figure 4c).

### 3.6 | Combination of colophonium and fragrance mix II improved mouse model outcomes

To assess in vivo impacts on a mixed culture, mice were exposed to a 1:1:1 mixture of 10^5^ colony forming units each of *Rm*HV, CONS-HV and *Sa*AD for 2 days following the induction of dermatitis (Figure 4d). Immediately after each bacterial inoculation, mice were treated with topical petroleum jelly (vehicle control) or the patch test reagents containing either paraben mix or an equal mixture fragrance mix II with colophonium (Figure 4d). The topical chemicals were applied to the ears the same day as the bacterial mix to prevent the differing growth curves of *Staphylococcal* spp. and *R. mucosa* from impacting microbial balance before topical exposure.^20^ The combination of fragrance mix II and colophonium created significant improvement in ear thickness (Figure 4e), inflammation (Figure 4f) and bacterial growth (Figure 4g) compared to the petroleum jelly vehicle or paraben mix. Paraben mix trended towards worsened outcomes and dysbiotic bacterial growth balance (Figure 4e–g).

### 3.7 | Targeted combination of patch test standards shifted microbiome in healthy controls

**FIGURE 5:**
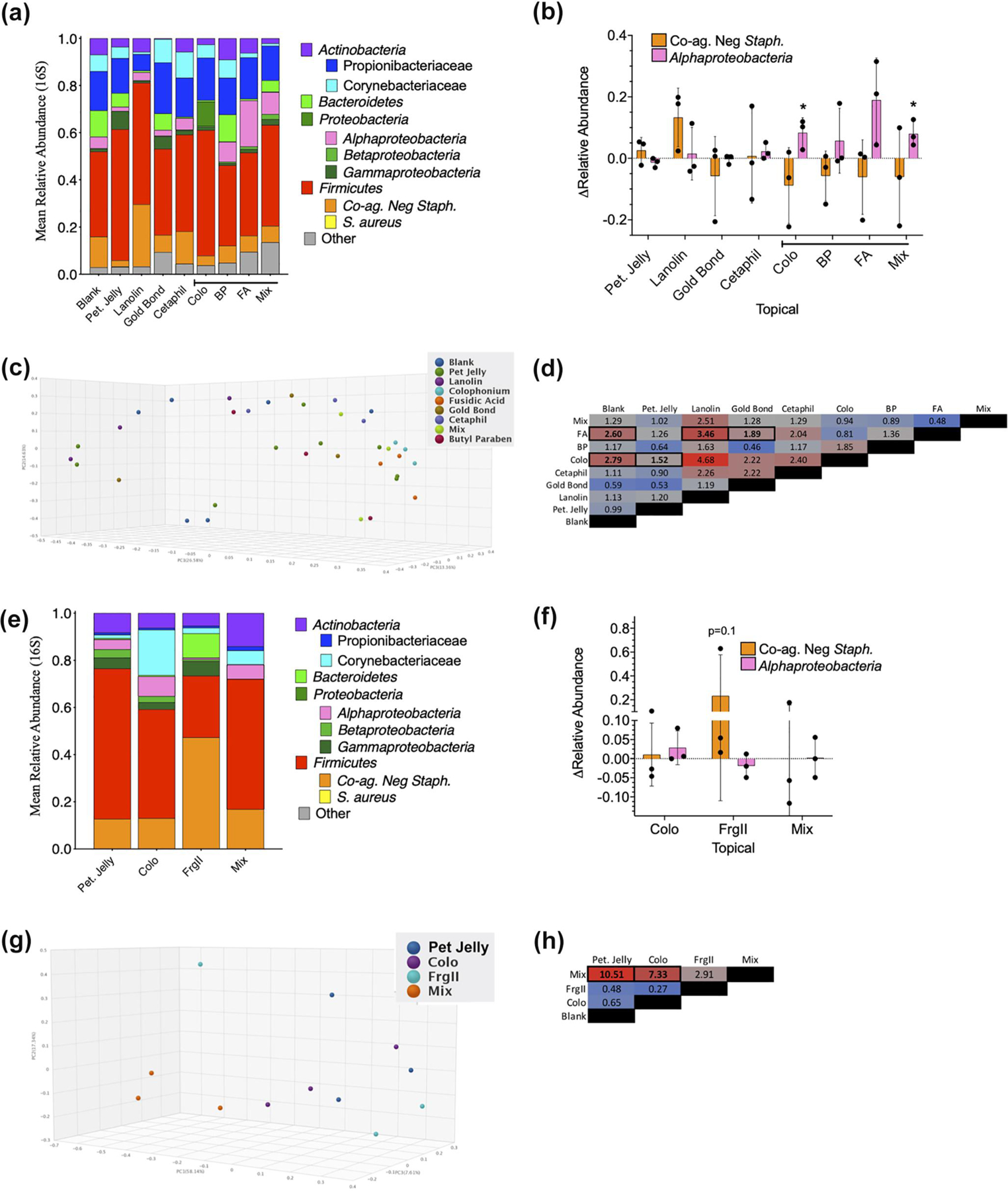
Topical exposure via patch testing proof-of-concept study impacts skin microbiome. (a) 16S for combined (*n* = 3) proof of concept for topicals indicated (petroleum jelly [Pet Jelly]; fusidic acid [FA] 453 μg/ml; colophonium [Colo] 833 μg/ml; butyl paraben [BP] 3.3 pg/ml; Mix, FA 453 μg/ml, Colo 833 μg/ml and BP 3.3 pg/ml—as tested in mice in Figure 3). (b) Per individual values for change in relative abundance of coagulase-negative *Staphylococcus* spp and *Alphaproteobacteria*, versus blank patch control. (c) 3D principal component analysis (PCA) plot using Bray–Curtis method. (d) Beta diversity statistic across indicated comparisons—bold and outlined boxes indicate *p* < 0.01. (e) Bacterial identification via metagenomic assessment for combined (*n* = 3) proof of concept for patch test supplies indicated (Pet Jelly; Colo 20% w/v; fragrance mix II (FrgII) 14% w/v, or Mix = Colo and FrgII mixed 1:1—as tested in mice in Figure 4). (f) Per individual values for change in relative abundance of Coagulase Negative *Staphylococcus* spp and *Alphaproteobacteria*, versus Pet. Jelly patch control. (g) 3D PCA plot using Bray–Curtis method. (h) Beta diversity statistic across indicated comparisons—bold and outlined boxes indicate *p* < 0.01. * = *p* < 0.05 calculated by AVONA with Bonferroni’s adjustment versus null impacts unless indicated and displayed as mean ± SD

In a small (*n* = 3) proof-of-concept study, 16S and ITS microbiome assessments were performed on healthy controls after exposure to colophonium, fusidic acid, butyl paraben or an equal mix of all three in dermatologic patch testing. Although we were unable to assess impacts on *S. aureus* given that none of our healthy controls were colonized, exposure to the combination of colophonium, fusidic acid, butyl paraben was associated with an enrichment of *Alphaproteobacteria* but generated no significant changes in coagulase-negative *Staphylococcus* spp. (Figure 5a,b); species level identification for *Roseomonas mucosa* is currently limited by gaps in 16S databases. Principal component analysis indicated significant differences in beta diversity for blank wells compared to both colophonium and fusidic acid (Figure 5c,d), but no changes in Shannon index alpha diversity for any group were seen (Figure S2a). In a second proof-of-concept study (also *n* = 3), metagenomic sequencing revealed that colophonium, alone and in combination with fragrance mix II was associated with an enrichment of *Alphaproteobacteria* in group-wise (Figure 5e) but not in paired analysis (Figure 5f). Fragrance mix II was associated with an expansion of coagulase-negative *Staphylococcus* spp. in group-wise analysis (Figure 5e) and trended towards similar impacts in paired analysis (Figure 5f). The mixture of colophonium and fragrance mix II trended towards altered beta diversity versus the petroleum jelly control (Figure 5g,h) without impacting alpha diversity (Figure S2b). No Food and Drug Administration (FDA) approved products were available for testing citral in isolation.

Although we were unable to perform culture-based assessment of the impact of topical products on fungal commensals, ITS analysis identified that the fungal class most associated with AD pathology, *Malasseziomycetes*, was also inhibited by the combination of colophonium, fusidic acid, butyl paraben in both group-wise and paired analysis (Figure S2c,d). Fragrance mix II and colophonium, however, did not significantly impact the relative abundance of these fungal sequences on metagenomic analysis (Figure S2e,f).

## 4 | DISCUSSION

While the effects of prescription antibiotics on the development of non-communicable diseases are well established,^21^ our findings indicate that the concern for anti-microbial impacts should also include common topical exposures. Our work presents an initial attempt to assess the antibiotic properties of the numerous topical skin care products. Although not all market-approved chemicals are common topical exposures, the US Environmental Protection Agency’s 2016 Chemical Data Report indicated that 8707 chemicals are on the US commercial market.^22^ In addition, although the bacterial isolates used in this study have been representative of the AD phenotype in multiple as-says,^4–6^ our in vitro analysis was limited to 12 total bacterial isolates from only four of the estimated 500–1000 different bacterial species on the skin.^23^ Therefore, the logistical limitations of testing numerous chemicals (at varying doses and combinations) against all available microbial isolates may be beyond current technologic capabilities. Thus, our patch test approach may offer a feasible means of identifying products with the potential to alter the skin’s microbial balance. Previous work has demonstrated that some ingredients from topical products can accumulate and persist on the skin in ways that impact the metabolic and bacterial sequence diversity of the skin microbiome.^14^ Our work provides additional insights by allowing for multiplexing both single ingredients and complex mixtures while using each participant as their own temporal-spatial control. However, our approach would require modification to assess the impacts of chronic exposures to the products of interest.

Yet even with the limited number of isolates and chemicals, our approach successfully identified topical ingredients that could positively influence growth of microbial cultures and enhance the microbe-specific outcomes in an established mouse model of AD. Although more nuanced than our culture-based assay, our patch test system was successful at identifying shifts in the microbiome in a small number of healthy controls. In the United States, follow up studies in patients with AD or those colonized with *S. aureus* may require FDA authorization and/or good manufacturing practice compliant production of our suggested formulations for over-the-counter sales. Inclusion of fusidic acid in any such formulation would carry regulatory limits in both North America and Europe^24^; furthermore, fusidic acid has not shown therapeutic benefit in clinical trials unless combined with topical steroids,^25^ thus making it a poor candidate for monotherapy. Inclusion of butyl paraben may be limited by consumer concerns for adverse effects.^26,27^ In contrast, both colophonium and citral are plant-derived compounds^28^ with historical use as anti-inflammatory, anti-pruritic and anti-microbial agents.^19,29–31^

The combination of lemon myrtle and colophonium had a net beneficial effect despite lemon myrtle alone having a more inhibitory effect on *Rm*HV than *S. aureus*. By using a net index that considered multiple isolates as the measure of success, we were able to find a combination that provided benefits beyond what could be found with the chemicals in isolation. This further highlights the need to screen products against multiple isolates as opposed to the current practice of limiting analysis to only *S. aureus*. However, given that commercial products which included citral and colophonium as ingredients had variable impacts on microbial growth, additional evaluation may be needed to optimize lemon myrtle and colophonium concentrations, as well as to identify other ingredients which might counter their beneficial effects. As demonstrated with our scratch assay results, compounds of interest should also be assessed for direct impacts on host cells given that these exposures could theoretically bolster or negate beneficial pre-biotic effects in a manner that is dependent upon the combination of chemicals used (Figures 3f and 4c). The mechanism of the inhibition seen by butyl paraben, citral and fusidic acid (as well as the mechanism for the partial reversal when in combination with colophonium) remains to be elucidated.

The data for lemon myrtle differs from our work using fragrance mix II given that the human exposure data for fragrance mix II cannot exclude contributions of other ingredients such as farnesol.^32^ In addition, given the reported apprehension to chemical agents in the AD patient population,^11,12^ we opted to test colophonium over synthetics like bronopol or kathon CG but cannot exclude potential in vivo benefits of these ingredients. However, screening of both natural and synthetic topicals outside of those included in patch testing kits may provide additional optimization while reducing the risk of allergic contact dermatitis reaction in this at-risk population.^33^

Our previous mechanistic work has established a combination of glycerophospholipids, cholinergic agonists and flagella as central to the therapeutic benefits of *R. mucosa*.^5,6^ Therefore, future studies should also investigate the impact of topical chemicals on bacterial production of these metabolites. Similarly, investigation is warranted for into whether skin care products influence the expression levels of any of the identified *S. aureus* virulence factors which contribute to AD pathology, such as delta-toxin or phenol-soluble modulins.^34^ Metabolomic evaluations of isolates exposed to varying sub-lethal concentrations of skin care products will elucidate impacts of these chemical exposures on microbial physiology. However, given that we did not identify any clear correlation between chemical structure and activity against the growth of the selected isolates (Figure S1), future targeted topical design is likely to require direct testing of ingredients rather than being able to predict outcomes based on chemical properties.

Given that some of the chemicals tested are also used in food production and may contaminate drinking water, the potential for inducing dysbiosis should also be investigated for gut or oral organisms.^35^ Even though our results support the expressed concerns over a potential role of topical chemicals in dysbiosis, our work does not establish a causal link between these environmental chemicals and AD pathogenesis. Our findings will require adequately powered follow up studies prior to formally recommending the avoidance of any specific products or ingredients. Overall, our findings begin to elucidate the role for topical exposures in the generation or prevention of dysbiosis even if it does not present a definitive ‘best recipe’ for topical product formulation. Future clinical validation may further aid patients and families struggling with AD by clarifying the targeted combinations of topical ingredients with pre-biotic effects towards beneficial commensals.

## Supplementary Material

Supplemental Figures

## Data Availability

All data is contained in the manuscript.

## What is already known about this topic?

- The microbiome is increasingly recognized as both a significant contributor to atopic dermatitis (AD) pathology and a potential therapeutic target.
- Nearly all the skin microbiome studies to date have asked participants to avoid topical products for the preceding days to weeks prior to sample collection.
- Patients and families struggling with atopic dermatitis (AD) have documented concerns for a contributory role of skin care products in AD pathology.

## What does this study add?

- Identifies chemicals that foster the growth of AD-associated bacterial isolates and thus may contribute to clinical dysbiosis.
- Demonstrates multiplex approach for testing chemicals using each participant as their own temporal-spatial control.
- Using a combination of culture-based, mouse model, and genetic microbiome assessment in the dermatologic patch test system we identified select chemicals which could offer pre-biotic effects for health-associated strains of commensal bacteria.

## What is the translational message?

- Our multiplex approach could be directly translated to current dermatologic research protocols to screen individual topical product ingredients and complex combinations.
- Continued optimization would elucidate the knowledge gaps in how skin care product choice influences AD through dysbiosis.
- Addressing patient concerns for the role of skin care product impact in AD pathology can be performed with an objective approach and with a goal of improving product choice.
