# Supplementary material for: Assessing the effects of common topical exposures on skin bacteria associated with atopic dermatitis
Source: Skin Health Dis. 2021 May 7;1(3):e41. doi: 10.1002/ski2.41 (PMC8555759; doi:10.1002/ski2.41)
Supplement: Supplementary file 1 — Supplementary Material [file SKI2-1-e41-s002.docx]

**Methods:**

*Bacterial Collection and Identification:*

Bacterial isolates were collected as previously described under the IRB approved protocol after written and oral consent was provided^20^. Briefly, two FloqSwabs (Copan, Brescia, Italy) moistened in sterile phosphate buffered saline (PBS; Corning Cellgro, Corning, NY) were rubbed on the subject’s skin at the antecubital fossa and volar forearm vigorously for 15-30 seconds. For patients with atopic dermatitis, sampling was done at these sites from affected lesional skin if present. One swab was placed into a 15mL conical tube (Corning Life, Corning, NY) with 2mL of sterile Hank’s balanced salt solution (HBSS; Sigma-Aldrich) containing vancomycin (300ug/mL) and amphotericin B (5ug/mL; Sigma-Aldrich, St. Louis, MO) to inhibit growth of Gram-positive bacteria and fungi. The remaining swab was placed into a 15mL conical tube containing 2mL of R2A (Reasoner's 2A) broth (Teknova, Hollister, CA) with similar concentrations of vancomycin and amphotericin B. The tubes, with swabs left in place, were then incubated at 32**°**C with constant shaking for 48-72 hours before plating 100uL from each tube onto an R2A agar plate (Remel, Lenexa, KS). Colonies were then taken for species identification by mass spectrometry using matrix-assisted laser desorption/ionization-time of flight (MALDI-TOF) analysis. Bacterial protein extraction for MALDI-TOF MS using the BioTyper (v3.1, Bruker Daltonics Inc., Billerica, MA) was performed by the NIH Clinical Center microbiology lab using previously described methods ^36^, instrument settings and calibration ^37,38^. BioTyper identification was supplemented by additional mass spectra profiles provided by several NIH developed databases^36,39,40^. All *R. mucosa* isolates used for subsequent studies were verified by MALDI-TOF analysis. Isolates were selected for topical exposure testing based on their impact in in vitro models of AD^4^, mouse models^4^, and based on the therapeutic benefits seen in clinical trials^5,6^.

*Chemical selection and testing*

Chemicals were chosen to form a broad representation of exposures that AD patients might encounter. Several common bathing therapies for AD were tested, including seawater, Dead Sea salt solution, and citric acid baths ^16^. A variety of parabens, a common preservative, were tested as well as the Chemotechnique NAC-80 patch test kit. The patch test kit features a range of common preservatives, additives and other chemicals meant to simulate typical environmental exposure levels.

The broth-soluble chemicals were dissolved in R2A or BHI broth media and diluted to the concentrations described in the literature. Chemicals tested include 480 mM NaCl, 54.14 mM MgSO4, parabens (methyl, ethyl propyl, butyl, benzyl), ZnO, Zn, soap, iodine, 5% Dead Sea salt solution, 0.005% bleach, and 75% ethanol as a control. In a 96-well plate, 100 µL of chemical was combined with 100 µL of 1/50 dilute bacteria. One row of wells was reserved for 200 µL of broth media and one row of wells was reserved for 100 µL of 1/50 dilute bacteria and 100 µL of broth media. The 96 well plates containing strains of *R. mucosa* were incubated at 32°C for 24 hours and 120 rpm shaking. The 96 well plates containing strains of *Staph spp.* were incubated at 37°C for 3 hours and 120 rpm shaking. Following the incubation period, absorbances were collected at 600 nm using a BioRad Benchmark Plus plate reader. Absorbance of the bacteria incubated with the chemical was compared to absorbance of the bacteria incubated in broth media and percent change of bacterial growth was calculated.

Chemicals insoluble in broth (Chemotechnique Diagnostic NAC-80 patch test kit and lotions) were incubated with bacteria on agar plates. 100 µL of 1/50 dilute *R. mucosa* was plated on R2A agar plates and 100 µL of 1/50 dilute *Staph.* spp was plated on Remel blood agar plates. Approximately 1 mL of chemical was applied to a round glass coverslip and placed in the center of the agar plate. Plates of *R. mucosa* were incubated at 32°C for 48 hours and plates of *Staph.* spp were incubated at 37°C for 24 hours. Following incubation, zone of inhibition was measured with Neiko digital calipers.

*Isolate selection and culturing*

3 Healthy Volunteer (HV) and 3 Disease Associated (AD) strains of *R. mucosa* were cultured in 5 mL Reasoner’s 2A (R2A) broth media for 24 hours at 32°C. 3 HV and 3 AD strains of *Staph* spp*.* were cultured in 5 mL Blood Heart Infusion (BHI) broth media for 24 hours at 37°C. The cultured bacteria were vortexed and diluted to 1/50 in 5 mL broth media. Selection was as previously described ^4,5^.

*Growth index calculation*

To quantify the chemicals’ effect on microbial growth balance, a growth index was created. Rather than measuring *R. mucosa* or *Staph.* spp growth individually, it synthesizes them into a measurement that assesses how the chemical might affect conditions for growth on the skin. The growth index aggregates R. mucosa and Staph spp. growth measurements (either zone of inhibition or percent growth) and calculates the overall balance between Gram- and Gram+ growth. A negative growth index value denotes conditions where *Staph. spp* is able to outcompete *R. mucosa*, and vice versa for a positive growth index value.

*Mix derivation*

Colophonium was ground into powder using a Qiagen TissueLyser LT and steel bead.  Colophonium powder, fusidic acid and butyl paraben were dissolved in molecular biology-grade distilled H2O and mixed with Cetaphil, Gold Bond lotions, or Vaseline in a Fisher Scientific Bead Mill 4 for 90 seconds at speed 5. Lemon myrtle oil (Tea Tree Therapy; Ventura, CA) was mixed into indicated products by stir.

*Patch testing*

Six total healthy volunteers were enrolled in an IRB approved study NCT03921515 and exposed to the topical chemicals via patch testing. Patch testing was used because the commercially available systems represent real-world concentrations for each product as used in skin care products. After mixing with Vaseline as above, 12 total wells of a Finn Chamber patch test (SmartPractice; Phoenix, AZ) were used. Three wells were Vaseline diluent alone, two were blank, the remaining wells contained the indicated chemical. Each location was randomized so that the relative positions of each chemical to each other was different for each participant. Comparisons between diluent control and chemical infused patches were made via 16S and ITS analysis. Each participant was instructed to avoid al topical skin care products for the 24 hours before, and during the patch testing experiment.

*Microbial DNA extraction from human skin*

Microbial DNA was extracted from the host as previously described ^6^, and extracted using the Qiagen DNA Microbiome kit (Hilden, Germany) using the Qiagen QIAcube as per manufacturer instructions. Isolated genomic DNA was quantified with Qubit 2.0 DNA HS Assay (ThermoFisher, Massachusetts, USA).

*Targeted microbiome analysis*

Library prep and sequencing were performed by CosomosID (Rockville, MD, USA). For 16S V1-V3, libraries were prepared using the Illumina 16S Metagenomic Sequencing kit (Illumina, Inc., San Diego, CA, USA) according to the manufacturer`s protocol. The V1-V3 region of the bacterial 16S rRNA gene sequences were amplified using the primer pair designed to amplify that specific region with Illumina adapter overhang nucleotide sequences at 5’ end .  The full-length primer sequences are:

27F: 5’ TCGTCGGCAGCGTCAGATGTGTATAAGAGACAG‐[ AGAGTTTGATCCTGGCTCAG]
534R: Reverse overhang: 5’ GTCTCGTGGGCTCGGAGATGTGTATAAGAGACAG‐[ ATTACCGCGGCTGCTGG]

Amplicon PCR was performed to amplify target gene out of input DNA templated from each respective sample. Briefly, each 25 μL of polymerase chain reaction (PCR) reaction contains 12.5 ng of sample DNA as input, 12.5 μL 2x KAPA HiFi HotStart ReadyMix  (Kapa Biosystems, Wilmington, MA) and 5 μL of 1 μM of each primer. PCR reactions were carried out using the following reaction conditions: an initial denaturation step performed at 95°C for 3 min followed by 25 cycles of denaturation (95°C, 30 s), annealing (55°C, 30 s) and extension (72°C, 30 sec), and a final elongation of 5 min at 72°C in a thermal cycler. PCR product was cleaned up with Mag-Bind RxnPure Plus magnetic beads (Omega Bio-tek, Norcross, GA). A second index PCR amplification, used to incorporate barcodes and sequencing adapters into the final PCR product, was performed in 25 μL reactions, using the same master mix conditions as described above. Cycling conditions were as follows: 95°C for 3 minutes, followed by 8 cycles of 95°C for 30”, 55°C for 30” and 72°C for 30”. A final, 5 minutes’ elongation step was performed at 72°C. The libraries were normalized with Mag-Bind EquiPure Library Normalization Kit (Omega Bio-tek, Norcross, GA) and then pooled. The pooled library was qualified and quantified using an Agilent 2200 TapeStation and sequenced (2x300bp paired-end read setting) on the MiSeq (Illumina, San Diego, CA). Because testing supplies were not sterile, sequencing of supplies was performed and those signatures were subtracted from the participant samples prior to analysis. The remaining reads in the samples were calculated for relative abundance and generation of 3D PCA plots were done by the CosmosID application.

For fungal diversity studies in our 50ng of isolated genomic DNA was used to amplify via PCR with proprietary primers (ITS1 and ITS2). All primers were synthesized by Integrated DNA Technologies (Coralville, IA, USA)) covering ITS1 and ITS2 regions. Specific primer selection and design (Admera Health, LLC, South Plainfield, NJ ) were chosen to achieve comprehensive taxonomic coverage, elimination of spike-in to gain maximal data. Final library quantity was assessed by Qubit 2.0 (ThermoFisher, Massachusetts, USA) and quality was assessed by TapeStation D1000 ScreenTape (Agilent Technologies Inc., California, USA).

Sequences of both the ITS primers used are as follows: ITS1-F ACCTGCGGARGGATCA; ITS1-R GAGATCCRTTGYTRAAAGTT; ITS2-F GTGAATCATCGARTCTTTG; ITS2-R TCCTCCGCTTATTGATATGC

Illumina® 8-nt dual-indices were used. Equimolar pooling of libraries was performed based on QC values and sequenced on an Illumina MiSeq V2 (Illumina, California, USA) with a read length configuration of 2x250bp.

*Mouse model*

Female or male mice age 6-8 weeks (age and sex matched within each experiment) were treated in the MC903 model as previously described ^5^. MC903 was applied to both ears on days -10 to 0, when applied 10e4 colony forming units of USA300LAC strain of *S. aureus* or of HV1 strain of *R. mucosa* were applied to both ears on days 0 and 1, then a PBS solution containing lemon myrtle oil (1% v/v in water) with colophonium (5% w/v) was applied to both ears on days 1 and 2. Ear were imaged and harvested on day 7. For experiment using a combination of bacterial isolates. MC903 model dermatitis was induced as before. For two consecutive days (day 0-1) mice were given a topical application of a 1:1:1 mixture of 5e5 CFU of *Rm*HV, CONS-HV, and *Sa*-AD immediately followed by application of Vaseline, a mixture of the patch test reagents for fragrance mix II and colophonium, or the patch test paraben mix. Ear were imaged and harvested on day 5.

*Scratch assay*

Scratch assay was performed as previously described using the HaCaT keratinocyte cell line purchased from American Tissue Culture Collection (ATCC; Manassas). Experiments were performed as previously described. Briefly, 100,000 cells were seeded in 24 well plates and allowed to adhere to the culture plate overnight. Cells challenged with bacteria were stimulated overnight prior to scratch; cells challenged with chemicals were stimulated 4 hour prior to scratch using the Autoscratch (BioTek; Winooski, VT). Cells were placed in the Cytation 5 (BioTek) at 37^O^C with 5% CO2; images and quantitation were performed by the Scratch App (BioTek).
