# Supplementary material for: Assessing the effects of common topical exposures on skin bacteria associated with atopic dermatitis
Source: Skin Health Dis. 2021 May 7;1(3):e41. doi: 10.1002/ski2.41 (PMC8555759; doi:10.1002/ski2.41)
Supplement: Supplementary file 2 — Supplementary Material [file SKI2-1-e41-s001.pdf]

Supplemental Figure 1

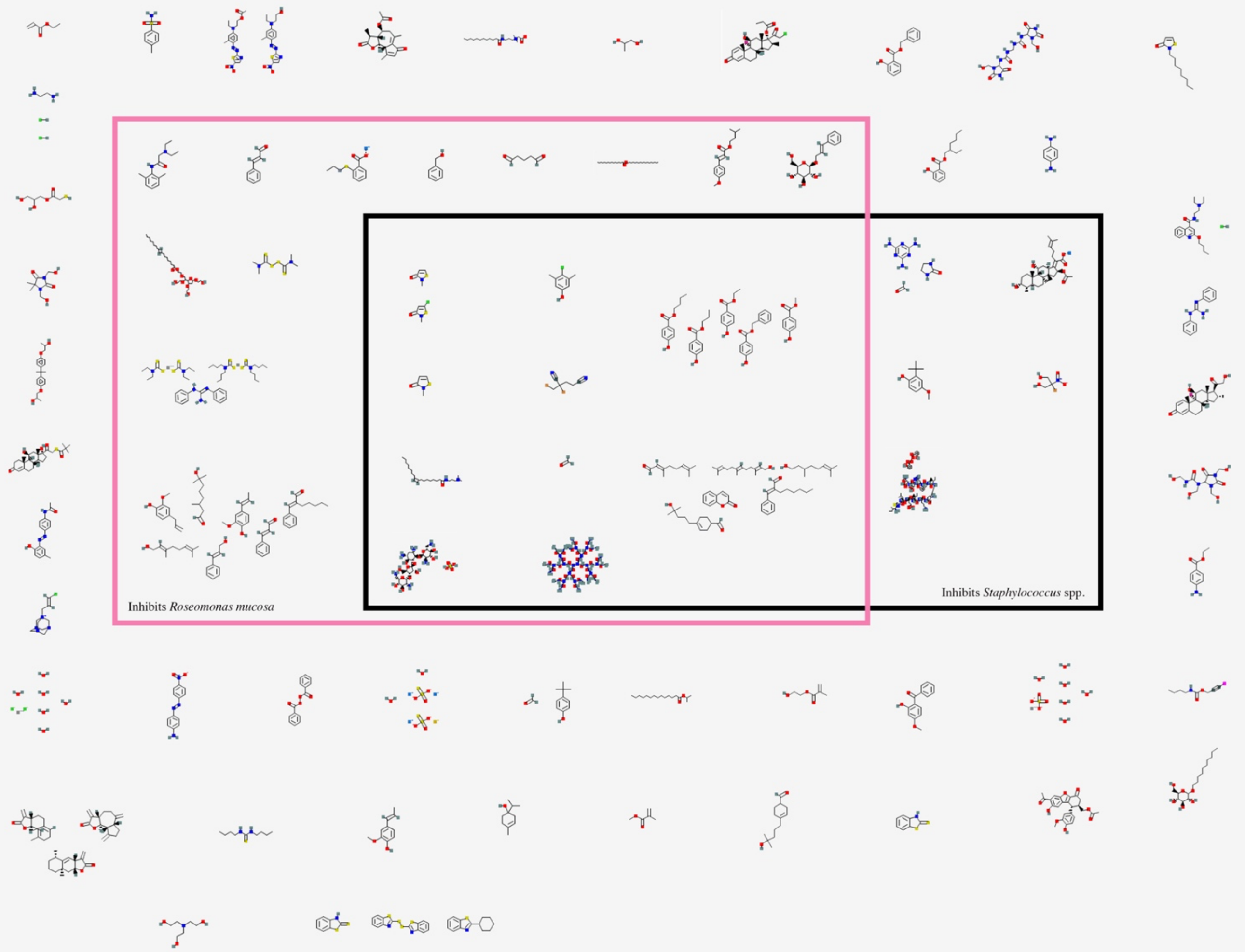

Supplemental Figure 1. Summary of the NAC80 chemical structures. Chemical structures from NAC80 tested chemicals sorted by whether they inhibit *R. mucosa* (inside pink box), *Staphylococcus* spp (inside black box), both (inside overlap), or neither (outside both boxes). Structure images pulled from PubChem.

Supplemental Figure 2

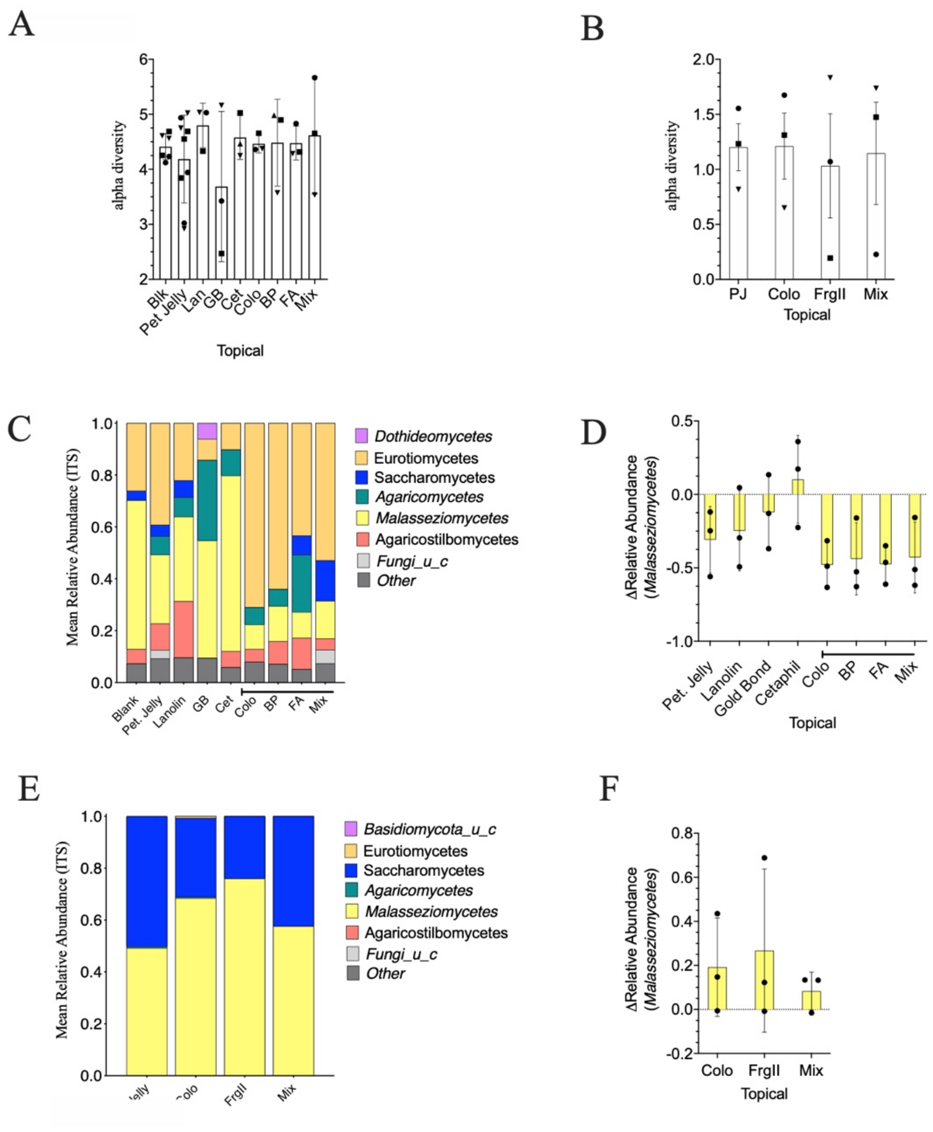

Supplemental Figure 2. Topical exposure impacts mycobiome in patch testing proof-of-concept. (A) Shannon diversity index for indicated groups in patch test for 16S analysis as in Figure 5A. (B) Shannon diversity index for indicated groups in patch test for bacterial metagenomic analysis as in Figure 5E. (A-B) Different shapes indicate samples taken from different volunteers. (C) ITS sequencing for combined (n=3) proof of concept for topicals indicated (Pet Jelly = petroleum jelly; FA, fusidic acid 453 µg/mL; Colo, colophonium 833 µg/mL, BP, butyl paraben 3.3 pg/mL; Mix, FA 453 µg/mL, Colo 833 µg/mL, and BP 3.3 pg/mL. (D) Per individual values for change in relative abundance of *Malasseziomycetes*, versus blank patch control. (E) Fungal identification via metagenomics assessment for combined (n=3) proof of concept for patch test supplies indicated (Pet Jelly = petroleum jelly; FA, fusidic acid; Colo, colophonium, Mix, Colo and FA mixed 1:1). (F) Per individual values for change in relative abundance of *Malasseziomycetes* versus Pet. Jelly patch control. Data is displayed as mean ± SD.
