# Supplementary material for: Assessing the effects of common topical exposures on skin bacteria associated with atopic dermatitis
Source: Skin Health Dis. Author manuscript; Available in PMC 2021 Oct 29. (PMC8555759; doi:10.1002/ski2.41)

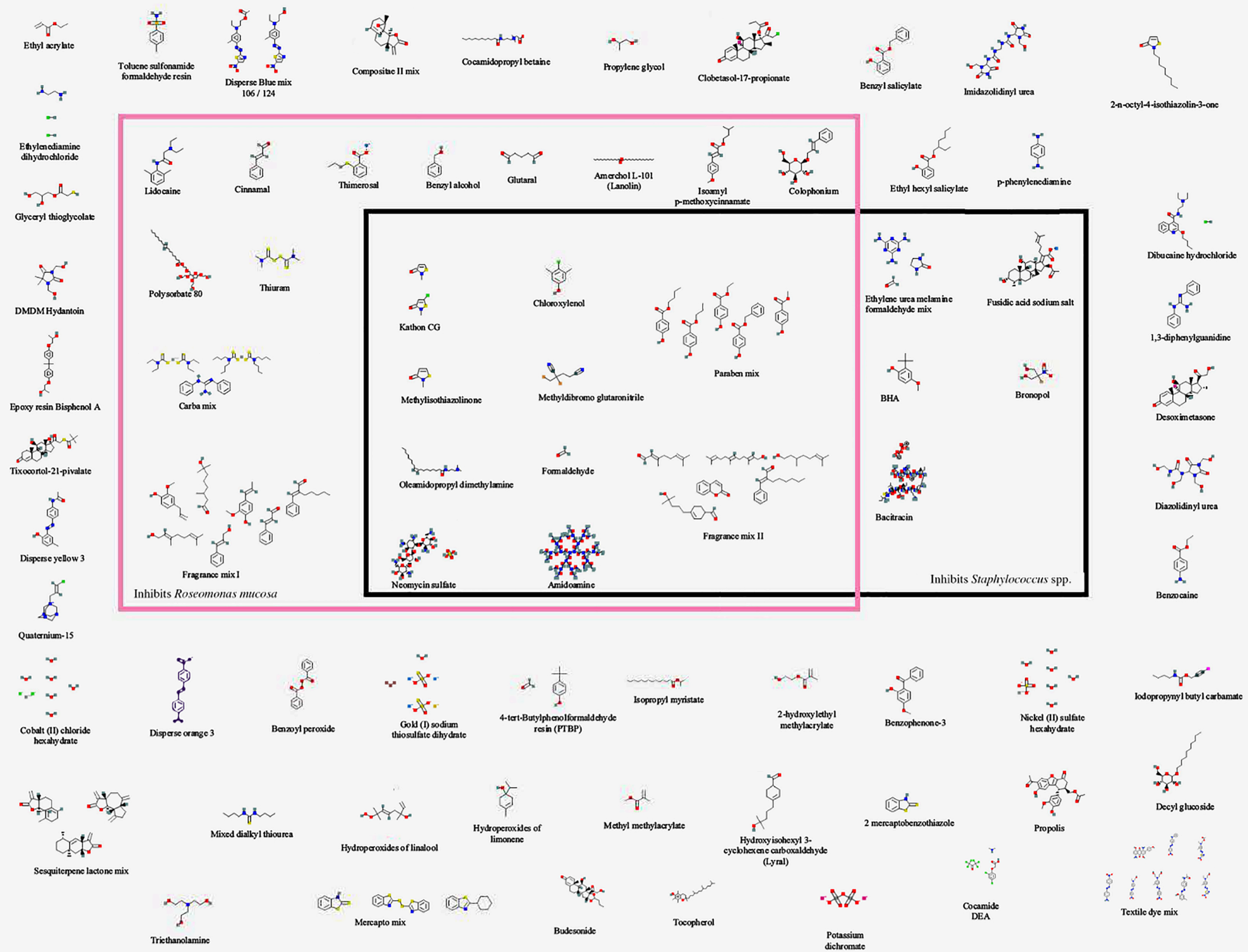

Ethyl acrylate
Toluene sulfonamide formaldehyde resin
Disperse Blue mix 106 / 124
Compositae II mix
Cocamidopropyl betaine
Propylene glycol
Clobetasol-17-propionate
Benzyl salicylate
Imidazolidinyl urea
2-n-octyl-4-isothiazolin-3-one
Ethylenediamine dihydrochloride
Lidocaine
Cinnamal
Thimerosal
Benzyl alcohol
Glutaral
Amerchol L-101 (Lanolin)
Isoamyl p-methoxycinnamate
Colophonium
Ethyl hexyl salicylate
p-phenylenediamine
Glyceryl thioglycolate
Dibucaine hydrochloride
Polysorbate 80
Thiuram
Kathon CG
Chloroxylenol
Paraben mix
Ethylene urea melamine formaldehyde mix
Fusidic acid sodium salt
1,3-diphenylguanidine
DMDM Hydantoin
Carba mix
Methylisothiazolinone
Methyldibromo glutaronitrile
BHA
Bronopol
Epoxy resin Bisphenol A
Desoximetasone
Tixocortol-21-pivalate
Oleamidopropyl dimethylamine
Formaldehyde
Fragrance mix II
Bacitracin
Diazolidinyl urea
Disperse yellow 3
Fragrance mix I
Neomycin sulfate
Amidoamine
Inhibits Roseomonas mucosa
Inhibits Staphylococcus spp.
Benzocaine
Quaternium-15
Cobalt (II) chloride hexahydrate
Disperse orange 3
Benzoyl peroxide
Gold (I) sodium thiosulfate dihydrate
4-tert-Butylphenolformaldehyde resin (PTBP)
Isopropyl myristate
2-hydroxylethyl methylacrylate
Benzophenone-3
Nickel (II) sulfate hexahydrate
Iodopropynyl butyl carbamate
Sesquiterpene lactone mix
Mixed dialkyl thiourea
Hydroperoxides of linalool
Hydroperoxides of limonene
Methyl methylacrylate
Hydroxyisohexyl 3-cyclohexene carboxaldehyde (Lyral)
2 mercaptobenzothiazole
Propolis
Decyl glucoside
Triethanolamine
Mercapto mix
Budesonide
Tocopherol
Potassium dichromate
Cocamide DEA
Textile dye mix

Supplemental Figure 2

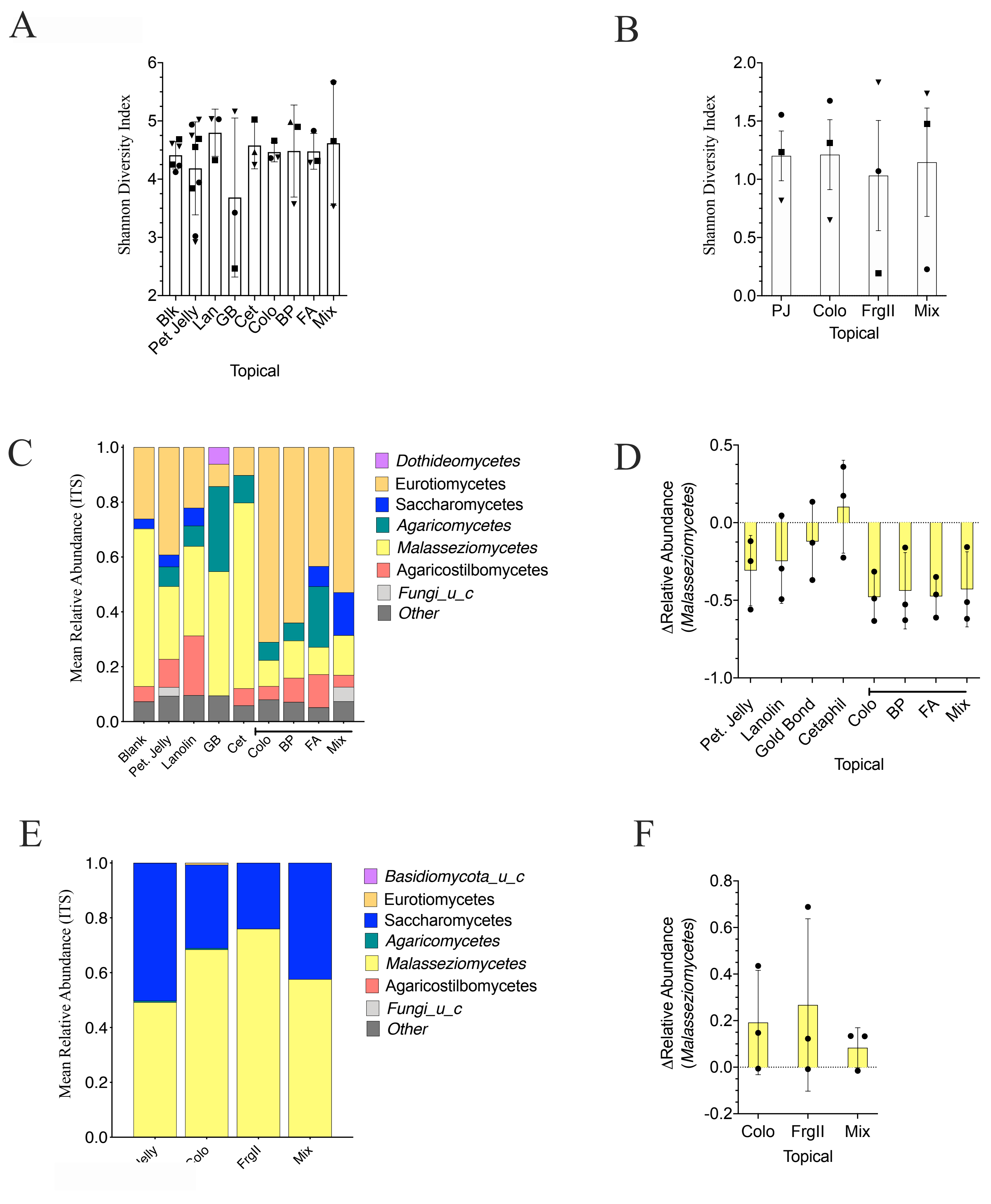

Supplement: Supplementary file 1 [file NIHMS1717841-supplement-Supplemental_Figures.pdf]
